# Perityphlitic Abscess*Read before the Buffalo Medical Association, September 2, 1879.

**Published:** 1879-10

**Authors:** Herman Mynter


THE BUFFALO

MEDICAL AND SURGICAL JOURNAL.

Vol. XIX.—OCTOBER, 1879.—No. 3.

### Original Communications.

### PERITYPHLITIC ABSCESS.*

BY HERMAN MYNTER, M. D.

A few months ago, Dr. E. R. Barnes read an able paper before the Buffalo Medical Club, on perityphlitic abscess and its surgical treatment, as advocated by the late Dr. Gurdon Buck, of New York. My attention was further directed to this subject by a case of perityphlitic abscess in my own practice that terminated favorably by adopting the treatment therein advocated. As very few had the opportunity to hear Dr. Barnes' paper, and as, in my opinion, this treatment ought to be studied and known by the profession generally, I take the liberty to present it to you this evening.

The terms typhlitis and perityphlitis,† the former signifying inflammation of the wall of the cæcum, the latter, inflammation in the tissues surrounding the cæcum, are generally applied to those cases in which there is perforation of the cæcum or the appendix vermiformis, and in which there occurs either acute

* Read before the Buffalo Medical Association, September 2, 1879.

† Bristowe, page 67.

peritonitis, or, provided the perforation takes place slowly, a local suppuration. Strictly speaking, the name is not a good one. It has been used to signify almost every variety of inflammation in the right iliac region. Bristowe believes that the majority of perforations occur in the appendix vermiformis, and sometimes in the cæcum itself, or the abscess takes its origin outside the wall of the cæcum. In a large and very valuable treatise on peritonitis from perforation of appendix vermiformis, by Professor With, of the University of Copenhagen, lately published, the author takes the same position. He calls these inflammations peritonitis appendicularis, on account of their origin from perforation of the appendix vermiformis sive appendicularis. Whatever may be the origin, we have a deeply-seated inflammation in the right ileo-cæcal region, often terminating in an abscess. The several writers make no difference in the symptoms of these abscesses, whether, on the one hand, they come from perforation of the appendix vermiformis or the cæcum, or, on the other, from perforation of that part of the ascending colon which is not covered with the peritoneum, or take their origin in the loose cellular tissue behind the cæcum or colon without perforation. My personal belief, on account of the anatomical relations, is, that there is some difference in the objective symptoms, as the abscess in one case is intraperitoneal, certainly for a time, in the other, extraperitoneal; being extraperitoneal, it will with greater facility extend downward in the loose cellular tissue behind the peritoneum.

Perityphlitis, to use that expression, is a very dangerous and not uncommon disease. According to Bristowe, it occurs after long-continued distension, or from ulceration, which so frequently attends distensions. Sometimes it may be caused by a simple perforating ulcer, or by the irritation and inflammation kept up by a foreign body; it may follow dysentery, typhoid fever, tuberculosis, or cancer; it may take its origin outside the cæcum or colon, either without any known cause (then often called rheumatic peritonitis), or on account of the inflammation extending to this spot from neighboring organs, as the liver, pleura, kidney, etc. If the abscess is the result of a perforation, we may find foreign substances, gallstones for instance, in the matter discharged. In thirteen cases published by Dr. Gurdon Buck, seven of them were from this cause, the foreign bodies consisting of fecal or calcareous matter from the size of a split pea to that of a date pit. In one case, eight or nine concretions were discharged through the incised wound. There can be no doubt that there is perforation of either cæcum or appendix vermiformis, when we find such fecal matter, and their frequency shows that the majority of these abscesses come from perforation. The abscesses often contain fetid smelling gases, as do all abscesses near the intestines. Perforation may take place directly into the peritoneal cavity, giving origin to a violent and fatally terminating peritonitis, or, if adhesions have taken place, resulting in an intraperitoneal abscess, walled in by adhesions. Such an abscess may again perforate the peritoneal cavity, or contiguous organs, as the colon, rectum, small intestines, bladder; or it may point towards the surface and, if no artificial opening is made, perforate in the iliac region. It may also perforate posteriorly; the abscess is then found in the loose cellular tissue behind the cæcum and ascending colon, although, I think, in such cases the abscess would be either the result of a rheumatic peritonitis, or of an inflammation in the neighboring organs. In this loose cellular tissue, the abscess may extend downwards to Poupart's ligament; behind it is found the strong iliac fascia, covering ileo-psoas muscle and crural nerve. This fascia will prevent the abscess from attacking the ileo-psoas muscle. The iliac fascia, as you remember, is adherent to Poupart's ligament in its whole length, except at the canalis cruralis, where the ligament goes in front of the vessels, the fascia behind. We have, therefore, here an opening through which an abscess coming down between the iliac fascia and the peritoneum, may appear on the femur and seek the surface.

A second direction such an abscess may take is down in the pelvis, and out through the apertura pyriformis. Lastly, the abscess may extend forwards in the loose cellular tissue between the bladder and the abdominal wall, and may here spread upwards towards the navel in the space between the plicae epigastricæ, or perforate into the bladder, or go through the inguinal canal down in the scrotum.

In regard to sex: ten of the cases gathered by Dr. Gurdon Buck were males and three females; of thirty patients, seen by Dr. With, twenty-three were males and seven females; my patient was a male. Of the forty-four here reported thirty-four were males, and ten females. The ages were different: below ten years of age, two; from ten to twenty years, ten; from twenty to thirty, fourteen; from thirty to forty, ten; from forty to fifty, eight.

The symptoms of perityphlitis are simply those of local and universal peritonitis. If perforation of appendix vermiformis takes place without adhesions having been formed, we shall often be unable to determine what the matter was before we make the post-mortem examination.

Numerous cases are on record, in which persons seemingly enjoying perfect health have been taken suddenly ill with universal peritonitis, and in which the post-mortem examination showed perforation of the processus vermiformis. In the cases where a local abscess is formed near the cæcum, the trouble may commence very suddenly with a chill and rigor, vomiting, accompanied with a deep-seated pain and tenderness in the right iliac region. Very soon a distinct fullness is felt here, and percussion may become dull. The fever is continuous, or slightly remittent, with exacerbations in the evening, but does not seem to reach a very high temperature. In the case treated by me the fever was generally 102 in the evening, 100 to 101 in the morning, and the pulse about 100. The pains and tenderness subside after a few days, and are only felt in the right iliac region, while at the commencement a soreness extends over the whole abdomen. The patients often complain of a feeling of deadness, and of slight œdema of the right leg, all resulting from pressure on the nerves and veins. Sometimes, especially in cases of universal peritonitis, the patients complain of dysuria, which may increase to perfect retention of the urine; jaundice also may occur. During the progress of the abscess the patient feels more and more prostrated; the fever continues, the fullness is more and more obvious, but fluctuation is very seldom felt early, only twice in the fourteen cases operated upon. The abscess may change its place, coming nearer and nearer to Poupart's ligament, the skin may become infiltrated and adherent, and at last fluctuation may be felt directly under the skin.

These inflammations, however, do not always give such serious symptoms or progress so far. It is often found that patients, suffering from such an abscess, formerly have had similar symptoms, which lasted only a few days and disappeared by rest and appropriate treatment, especially by the administration of opium. Professor With, in the treatise mentioned above, calls attention to these seemingly insignificant symptoms, and says that he has no doubt that these slight attacks of pain in the right iliac region, which are often treated as colics, very often indicate one of the most serious diseases, so much the more dangerous, as it may very often be misjudged in the commencement, when it is still possible to arrest it, or it may rapidly terminate in a universal peritonitis even in the healthiest and strongest subjects. That such slight attacks of inflammation around the processus vermiformis do occur frequently, more frequently indeed than is generally believed, is shown by the statistical examinations of another Danish physician, Dr. Toft, referred to in Professor With's treatise. In 300 post-mortem examinations he found the processus vermiformis normal 190 times, while it was more or less diseased 110 times, or showed traces of previous disease; therefore, Professor With says, in more than one-third of all corpses we may expect to find traces of disease of appendix vermiformis, while it is probable that they should have given some symptoms during life. Prof. With himself mentions one case in which adhesions and cicatricial tissue were found around the processus vermiformis by a post-mortem examination, which could be traced to a previous attack of perityphitis. He thinks, therefore, and his opinion is based upon a large hospital experience, that there is a gradual transition from the slight attacks, with pain and local symptoms of peritonitis, through the more or less severe forms to the fatally terminating peritonitis, from perforation of the processus vermiformis. It is the same disease in its different phases, which may be still more plainly shown by the fact that often slight attacks become, by injudicious use of cathartics, or by other injuries, the severe and fatal cases.

Professor With divides his cases, thirty in all, into three classes, each of which he considers as a stadium of the same disease, although they differ clinically very much. The first stadium he calls the adhesive, *peritonitis appendicularis adhæsiva*, when the ulceration progresses so far that the peritoneum is attacked and adhesions are formed. The second stadium, *peritonitis appendicularis localis*, is characterized by local peritonitis and primary abscess.

The third stadium is *peritonitis appendicularis universalis*, when we have diffuse peritonitis by perforation into the peritoneal cavity. Of cases of universal peritonitis he has seen and treated fourteen, of which twelve died and two recovered; eleven were males, and three females, the ages ranging between seventeen and forty-two years. In three of these cases there had (respectively two, three and ten years ago) formerly been symptoms of local peritonitis. The disease commenced suddenly in twelve of the fourteen cases, without previous admonition; in six of these it commenced as a local peritonitis, which afterwards became universal. The duration of the local stadium varied from four to fourteen days; of the universal, from two to six days.

The second stadium, *peritonitis appendicularis localis*, is represented in Professor With's treatise partly by six of the already mentioned fourteen cases of universal peritonitis, in which the disease commenced as a local peritonitis, afterwards becoming universal; partly by sixteen other cases, all of which recovered. In one of these he was able three years afterwards, when the patient died of some other disease, to show traces of perforation of the appendix vermiformis. To this stadium belong the thirteen cases mentioned by Dr. Gurdon Buck, and also the case I treated. Nine of the sixteen cases treated by With had suffered formerly from one or more similar attacks of shorter duration, as also had my patient.

In eight of With's sixteen cases, in which the symptoms were lighter, he found pain and resistance in the right iliac region, but besides that the abdomen was normal. In the other more severe eight cases, the whole abdomen was more or less painful and distended, but especially painful in the right iliac region. In five cases a smaller tumor was felt, in five others a larger; all patients had generally high fever, with remissions in the morning, coated tongue, vomiting, no appetite and were sleepless.

Professor With calls those cases, in which the ulceration in the appendix vermiformis goes so deep that the peritoneal covering is affected, *peritonitis appendicularis adhæsiva.* He acknowledges that we cannot fix the diagnosis in these cases with absolute certainty; but, as he says, there are signs and warnings that one of the most dangerous diseases is developing, and if we do not take heed we may, in a few hours, have to treat a case of universal peritonitis. The symptoms, he says, may be studied in those cases which died either of universal peritonitis or had a local abscess, but formerly presented lighter, but similar symptoms. Nine of the sixteen cases formerly experienced attacks, either of a similar nature, more or less severe, or else had symptoms of some local trouble in the right iliac region. Therefore, he says, whatever may be the connection between these symptoms and our adhesive peritonitis, and although we must acknowledge that we can scarcely consider them all as the result of this, it is a fact that those symptoms are often found in patients, who afterwards have a perforation of the processus vermiformis; that they may precede the perforation but a short time, sometimes immediately, and that it would be foolish to ignore them, if we cannot give a sure proof that they are the direct result of the disease. Even if the patient has made a mistake in his diet, and the case is similar to that of a simple catarrh of the bowels, we must be suspicious if the pains are severe, attended with a little soreness and tension in the ileo-cæcal region, and perhaps a little vomiting; and we must remember that a mistake in the diagnosis and treatment may be fatal, while a treatment with that possibility in view is without danger, and always alleviates the symptoms.

If we now consider the *diagnosis*, then this is not generally difficult, if we have the possibility of a perforation of the processus vermiformis in our mind. It may look like a simple colic or like a gallstone colic, especially if the pains commenced in the upper and right part of the abdomen and extend inwards toward the umbilicus and accompanied with vomiting.' But we shall soon discover that the attacks of pain are continuous, with but few and short remissions; that the soreness is most pronounced in the ileo-cæcal region; that the vomited matter is of greenish color, and that a tumor or a resistance will be felt in the iliac region; the fever, dysuria and pains in the right leg also are important diagnostic signs. If the patient be a female, we may think of metritis, parametritis and the different local diseases which may be found around the uterus during menstruation. Most difficult is, after With, the differential diagnosis of typhlitis stercoracea, and peritonitis appendicularis; the former developes more slowly with slight constipation, flatulence and a painless movable tumor, which is more in the direction of the cæcum and ascending colon; there is also a tendency to ileus.

If perforation of the cæcum occurs afterwards, we shall get a secondary perityphlitis with abscess in the iliac fossa, which has a deeper position, being behind the cæcum, and may extend downwards with greater facility on account of its position in the loose cellular tissue behind the peritoneum.

We now come to consider the most important subject—the treatment of these troubles. Here the course advocated by Professor With is just as important as that advocated by Dr. Gurdon Buck, or perhaps more important, inasmuch if we follow it we shall have less opportunity of resorting to Gurdon Buck's operation. With's treatment is told in a few words: no cathartics at all, absolute rest, opium, leeches, poultice and fluid diet. He warns most decidedly against the use of cathartics, and points out that the twelve patients who died of universal peritonitis had all been treated at home with castor oil or clysmata, while of the two patients who had universal peritonitis, but recovered, cathartics were not used at all in one case, while in the other, a single dose of castor oil was administered at home, which augmented considerably the pain. Of fourteen other cases with local peritonitis, eleven were treated in the hospital without cathartics or clysmata, and they all recovered in an average of twenty-one days, while the disease lasted much longer in three cases, in which cathartics had been used. He first allows their use when all local symptoms have completely disappeared, and there is no danger of peristaltic movements loosening the adhesions. He seeks to avoid spontaneous movement of the bowels and peristaltic movement by the use of opium, advising ten to twenty drops of tincture of opium three times a day or oftener. Leeches, a light poultice, absolute rest, and a strict diet of iced milk, tea and thin oatmeal gruel, comprise the treatment.

In light cases the pain disappears by this treatment in five or six days, and after ten days the opium is discontinued, and the bowels will then move spontaneously in a few days. In more severe cases, he keeps his patients constipated till all local symptoms have disappeared. This generally occurs in about fourteen days. He uses the opium a few days longer, and after its discontinuance, spontaneous movement will soon occur, or, if there is tenesmus, by aid of an injection. Twenty-four days is the longest time he has kept his patients constipated, and he assures us that they suffer no inconvenience from it. If, in spite of the treatment, the abscess should progress and become superficial, and fluctuation be felt, incision is indicated.

Professor With's treatise, I regret, is written in a language understood by very few of the profession. It contains such a vast amount of valuable information in regard to this disease that it ought to become the common property of the profession. If we now compare the above with the treatment advocated by Dr. Gurdon Buck and supported by thirteen, or, by including my case, fourteen cases (there may be many more, but I have not been able to find them), we will observe a marked difference. He uses leeches and poultice, but he advises five to ten grains of calomel, to be followed by castor oil, and thereafter opium in such quantity as to maintain a moderate state of narcotism. After the lapse of one week, if there is no clear indication of resolution going on, he advises the operation without waiting for fluctuation. In the two cases which he himself treated, the pain increased after cathartics, and the patients felt worse. In the other eleven cases there is no record referring to the use of cathartics. In my case, the patient had taken a large dose of salts the day previous to his attack. He did not take any cathartics the first three days, but on the fourth small doses of castor oil and injection of catnip tea, as he was very much oppressed. The cathartic did not relieve him, and the fever increased. All fourteen cases were very severe.

The *operation* is performed in two ways. Parker's method consists in an incision from three to six inches in length across the tumor a little above and parallel with Poupart's ligament. The skin and subjacent tendinous and muscular layers are divided till the transversalis fascia is exposed. An exploring needle, or the trocar of an aspirator, is then inserted in search of matter, and when this is found, a free incision is made with the trocar as a guide. In two cases, after the exposure of the transversalis fascia, the wound was dressed open, and a spontaneous opening was formed on the second and third day.

Gurdon Buck's method is different. He does not make any incision, but inserts a sharp-pointed canula, after having, at most, first perforated the skin with a tenotome, and having found matter, he enlarges the puncture with the knife sufficiently to afford free outlet for the contents of the abscess, the canula acting as guide for the knife. He chooses the most prominent point of the tumor for the puncture, and considers it harmless, even if he should penetrate the intestines. In case of a failure to reach the collection of pus by a first attempt, a second introduction may be safely tried at another selected spot. By this method, he asserts, the subsequent liability to a hernial protrusion is prevented. The time, at which the operation has been performed in fourteen cases, was: seventh day, two cases; eighth day, one case, tenth day, five cases; eleventh day, two cases; twelfth day, one case; thirteenth day, one case; fourteenth day, one case; and in one case earlier than the seventh day.

Fluctuation was discovered in but two cases before the operation; he advises not to wait for fluctuation. His rule is to operate after the lapse of one week from the onset of the disease unless there should be clear indications of resolution going on. By this treatment, he says, we may be said to have disarmed this disease of its terrors.

It is scarcely possible, if we look at the favorable results obtained by Professor With, not to acknowledge that they are due principally to the omission of cathartics, and the prevention of peristaltic movements.

Twelve died of universal peritonitis, but they entered the hospital in an unfavorable condition, after having been purged at home; and of the two serious cases with universal peritonitis, which recovered, one had not taken cathartics at all, the other only one dose of castor oil. Sixteen cases recovered, many of them with very serious symptoms of local abscess. On the other side, it cannot be denied that some of the twelve cases might have recovered by Gurdon Buck's operation, as they had symptoms of local abscess before the universal peritonitis set in, in four cases respectively seven, nine, ten, and fourteen days. Neither can it be denied that operative treatment in other cases might have cut short the long convalescence, and would surely have prevented the danger of universal peritonitis from secondary perforation of the abscess, and probably the danger of a relapse in the future, which is always more or less probable. By following strictly the treatment proposed by Professor With, we shall have less opportunity of using Gurdon Buck's operation; but, on the other hand, a judicious use of this operation will considerably lessen the danger, if the abscess progresses in spite of the treatment, and will be likely to prevent relapses.

Allow me, lastly, to report the case I treated; not because it contains anything more remarkable than the other cases published, but simply because it illustrates the extreme value of Gurdon Buck's operation.

Mr. Charles Lautz, forty-one years of age, engaged in a large manufacturing business, always enjoyed good health until five years ago, when he had an affection of the liver, which at that time was considered to be gallstone colic. He has since had three attacks similar to the one which follows, one of which kept him in bed six weeks. Last year he had a very severe intermittent fever. May 7th, 1879, he retired to bed perfectly well, but was awakened at three o'clock in the morning with violent pains in the right ileo-cæcal region. The pains were followed with vomiting, chills, with elevated temperature, perspiration, and a feeling of extreme weakness. The day previous he had taken a dose of salts. When I saw him, in the course of one hour, the temperature was 102, pulse 106; used sulphat. morphine gr. ¼, hypodermically, and ordered hot fomentations to the abdomen.

May 8. The pains continued during the day in spite of frequent hypodermic injections of morphine. A slight resistance is felt in the right cæcal region, commencing about two inches below the ribs and extending downward to a plane through the anterior superior spinous process of the ilium. Percussion was dull above the swelling, soreness over the whole abdomen, but especially over the ileo-cæcal region. Ordered ten leeches upon the ileo-cæcal region.

May 9. Felt relieved after the leeches during the first part of the night, but towards morning the pains returned and continued during the day, although morphine was frequently used. Temperature 102, pulse 96. No vomiting, but hiccoughs. The swelling more distinct. The pains extend downward into the right leg. Considerable meteorrhismus and oppression. Repeat the leeches and castor oil.

May 10. Temperature 103, no action of bowels. Ordered two grains of calomel every two hours.

May 11. Several movements of the bowels. Temp. 102½.

May 13. Temperature 102, abdomen softer, except in the right ileo-cæcal region, where the swelling is more prominent; pains decreasing.

May 18. Temperature remains between 101 and 103; the tumor seems to extend farther downwards towards Poupart's ligament; cannot tolerate any pressure over the tumor; the rest of the abdomen normal. The bowels move spontaneously. Takes nourishment only in fluids. Drs. Miner and Tobie called in consultation.

May 20. Under chloroform narcosis; operation; Drs. Miner and Tobie being present, and assisting. An incision, three inches long, was made half an inch above Poupart's ligament, and parallel with it, through the skin and the different muscular layers till the fascia transversalis was reached. Even then no fluctuation was perceptible. A sharp-pointed canula, in connection with Dieulafoix aspirator, was thrust upwards and backwards, and having entered about one inch, matter flowed into the aspirator. A free incision was now made with the canula as guide, and about one-third pint of offensive matter was evacuated, but no concrements were found. The cavity was explored with the finger, and was found to extend upwards behind the cæcum and forwards toward the bladder. The cavity was syringed out with carbolized water, a drain-tube introduced and the wound bandaged with a ten per cent. solution of carbolic acid in sweet oil. The fever disappeared immediately, the appetite returned, the wound healed kindly, so that on the 31st of May, eleven days after the operation, the patient sat up.

June 5th. Wound almost healed; he is able to drive out; a small fistula, with occasional discharge of matter, kept open for fourteen days longer; he suffered for some time considerably from meteorrhismus and difficult digestion, but little by little this symptom disappeared, although he even now occasionally complains of it, and on the 20th of June he was discharged cured.

